# Supplementary material for: Combining Flow Cytometry and Metagenomics Improves Recovery of Metagenome-Assembled Genomes in a Cell Culture from Activated Sludge
Source: Microorganisms. 2023 Jan 10;11(1):175. doi: 10.3390/microorganisms11010175 (PMC9864227; doi:10.3390/microorganisms11010175)
Supplement: Supplementary file 1 [file microorganisms-11-00175-s001.zip › 03_Abdulkadir_FC_MAG_Supplementary_file_1_Figure_S1.pdf]

Combining Flow Cytometry and Metagenomics Improves Recovery of Metagenome-Assembled Genomes in a Cell Culture from Activated Sludge

Nafi'u Abdulkadir, Joao Pedro Saraiva, Florian Schattenberg, Rodolfo Brizola Toscan, Felipe Borim Correa, Hauke Harms, Susann Müller, Ulisses Nunes da Rocha

A

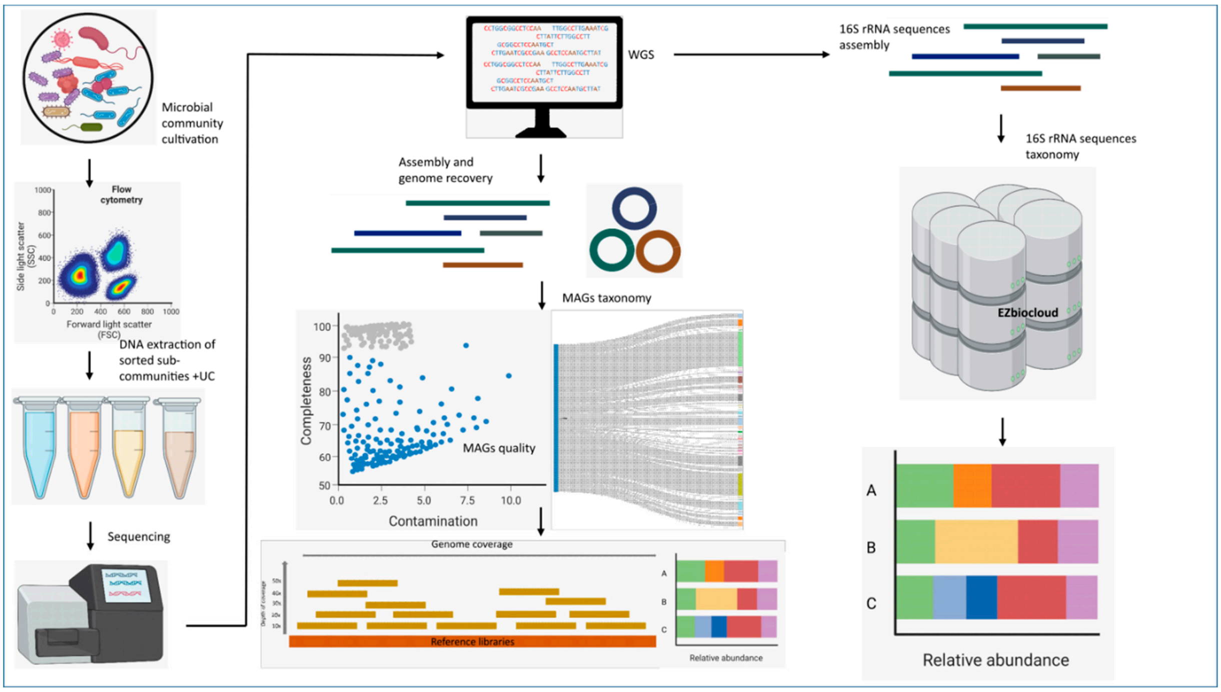

Combining flow cytometry and metagenomics improves recovery of metagenome-assembled genomes in a cell culture from activated sludge

Nafi'u Abdulkadir, Joao Pedro Saraiva, Florian Schattenberg, Rodolfo Brizola Toscan, Felipe Borim Correa, Hauke Harms, Susann Müller, Ulisses Nunes da Rocha

B

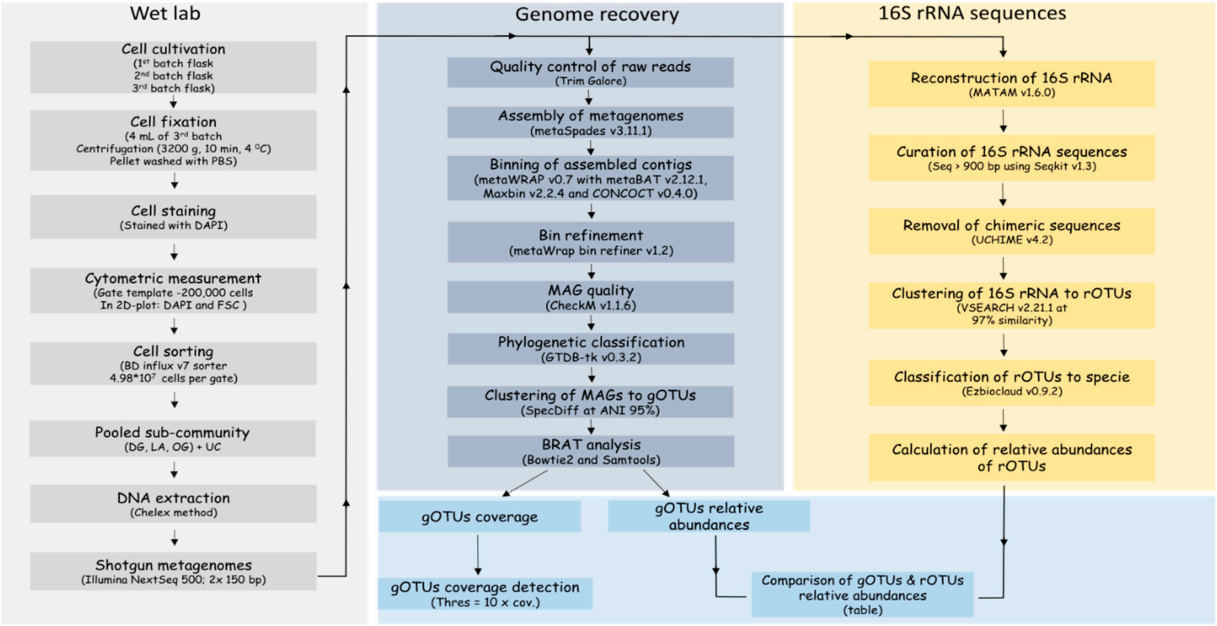

Supplementary file 1: Figure S1 – Study workflow. **(A)** Experimental design including microbial community cultivation, genome recovery and reconstruction of 16S rRNA sequences from metagenomes. **B)** Detailed overview of the tools/devices used. The experiment was run with cells of a microbial community from activated sludge of a wastewater treatment plant. Cells were cultivated in three batches, fixed and measured cytometrically to visualize community structure. Next, three different sub-communities were sorted: Dominant gates (DG), Low Abundant gates (LA) and Outer gates (OG). An Unsorted (UG) community was used as control. Next, DNA extraction and whole-genome sequencing for all groups was performed. MetaWrap [1] was used to recover all metagenome-assembled genomes (MAGs). CheckM [2] was to assess MAG quality. Taxonomic classification was determined using the genome taxonomy database (GTDB-tk) [3]. Genome coverage was determined by mapping the reads from the metagenomic libraries to the recovered MAGs using Bowtie2 [4]. MAG relative abundance was determined using SAMtools [5]. 16S rRNA sequences were reconstructed from metagenomes using a MATAM [6] pipeline and determined their phylogeny using EZbiocloud [7] database.

References

[1] Uritskiy, G.V.; DiRuggiero, J.; Taylor, J. MetaWRAP—a Flexible Pipeline for Genome-Resolved Metagenomic Data Analysis. *Microbiome* **2018**, *6*, 158, doi:10.1186/s40168-018-0541-1.

[2] Parks, D.H.; Imelfort, M.; Skennerton, C.T.; Hugenholtz, P.; Tyson, G.W. CheckM: Assessing the Quality of Microbial Genomes Recovered from Isolates, Single Cells, and Metagenomes. *Genome Res.* **2015**, *25*, 1043–1055, doi:10.1101/gr.186072.114.

[3] Chaumeil, P.-A.; Mussig, A.J.; Hugenholtz, P.; Parks, D.H. GTDB-Tk: A Toolkit to Classify Genomes with the Genome Taxonomy Database. *Bioinformatics* **2020**, *36*, 1925–1927, doi:10.1093/bioinformatics/btz848.

[4] Langmead, B.; Salzberg, S.L. Fast Gapped-Read Alignment with Bowtie 2. *Nature Methods* **2012**, *9*, 357–359, doi:10.1038/nmeth.1923.

[5] Li, H.; Handsaker, B.; Wysoker, A.; Fennell, T.; Ruan, J.; Homer, N.; Marth, G.; Abecasis, G.; Durbin, R.; 1000 Genome Project Data Processing Subgroup, The Sequence Alignment/Map Format and SAMtools. Bioinformatics 2009, 25, 2078–2079, doi:10.1093/bioinformatics/btp352.

[6] Pericard, P.; Dufresne, Y.; Couderc, L.; Blanquart, S.; Touzet, H. MATAM: Reconstruction of Phylogenetic Marker Genes from Short Sequencing Reads in Metagenomes. *Bioinformatics* **2018**, *34*, 585–591, doi:10.1093/bioinformatics/btx644

[7] Yoon, S.-H.; Ha, S.-M.; Kwon, S.; Lim, J.; Kim, Y.; Seo, H.; Chun, J. Introducing EzBioCloud: A Taxonomically United Database of 16S RRNA Gene Sequences and Whole-Genome Assemblies. *Int J Syst Evol Microbiol* **2017**, *67*, 1613–1617, doi:10.1099/ijsem.0.001755.
