# Supplementary material for: Combining Flow Cytometry and Metagenomics Improves Recovery of Metagenome-Assembled Genomes in a Cell Culture from Activated Sludge
Source: Microorganisms. 2023 Jan 10;11(1):175. doi: 10.3390/microorganisms11010175 (PMC9864227; doi:10.3390/microorganisms11010175)
Supplement: Supplementary file 1 [file microorganisms-11-00175-s001.zip › 04_Abdulkadir_FC_MAG_Supplementary_file_2_Figure_S2.pdf]

Combining Flow Cytometry and Metagenomics Improves Recovery of Metagenome-Assembled Genomes in a Cell Culture from Activated Sludge

Nafi'u Abdulkadir, Joao Pedro Saraiva, Florian Schattenberg, Rodolfo Brizola Toscan, Felipe Borim Correa, Hauke Harms, Susann Müller, Ulisses Nunes da Rocha

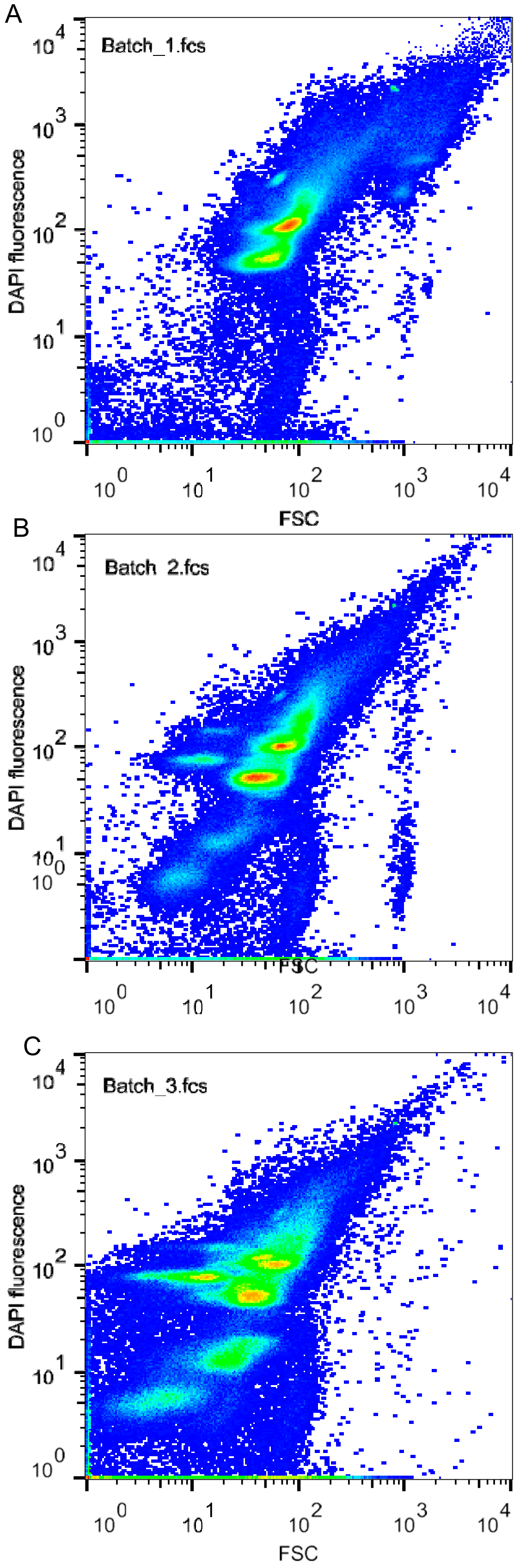

Supplementary file 2 Figure S2. Flow cytometry measurements of microbial communities based on forward scatter (FSC) and DAPI fluorescence. A) First batch; B) Second batch; C) Third batch of cultivation
