# Supplementary material for: Combining Flow Cytometry and Metagenomics Improves Recovery of Metagenome-Assembled Genomes in a Cell Culture from Activated Sludge
Source: Microorganisms. 2023 Jan 10;11(1):175. doi: 10.3390/microorganisms11010175 (PMC9864227; doi:10.3390/microorganisms11010175)
Supplement: Supplementary file 1 [file microorganisms-11-00175-s001.zip › 05_Abdulkadir_FC_MAG_Supplementary_file_3_Table_S1.pdf]

Combining Flow Cytometry and Metagenomics Improves Recovery of Metagenome-Assembled Genomes in a Cell Culture from Activated Sludge

Nafi'u Abdulkadir, Joao Pedro Saraiva, Florian Schattenberg, Rodolfo Brizola Toscan, Felipe Borim Correa, Hauke Harms, Susann Müller, Ulisses Nunes da Rocha

Supplementary file 3: Table S1. Gate clustering according to cell abundance and relative abundances. The average cell abundance per gate (7658.1 cells). The black line is the cut-off as in the section 2.5 of the main manuscript.

Sub-community	Gate [a]	Gate count [b]	Gate relative abundance (%) [c]	Cell count per sub-community [d]	Sub-community relative abundance (%) [e]
Dominant gates	G1	43129	21.56	116796	58.39
	G2	34745	17.37		
	G3	11728	5.86		
	G4	18548	9.27		
	G9	8646	4.32		
Low abundant gates	G5	550	0.28	44024	22.03
	G6	7311	3.66		
	G7	1619	0.81		
	G8	3272	1.64		
	G10	1115	0.56		
	G11	1525	0.76		
	G12	4305	2.15		
	G13	1494	0.75		
	G14	983	0.49		
	G15	4279	2.14		
	G16	2200	1.10		
	G17	4394	2.20		
	G18	3190	1.60		
	G19	634	0.32		
	G20	6498	3.25		
	G21	655	0.33		
Outer gates				39180	19.59

[a] The cluster of microbial cells that differ from other based on their optical properties

[b] The number of microbial cells in each gate

[c] The relative proportion of each gate in the whole community

[d] The total number of microbial cells per sub-community

[e] The relative proportion of sub-communities in the whole community
