# Supplementary material for: Combining Flow Cytometry and Metagenomics Improves Recovery of Metagenome-Assembled Genomes in a Cell Culture from Activated Sludge
Source: Microorganisms. 2023 Jan 10;11(1):175. doi: 10.3390/microorganisms11010175 (PMC9864227; doi:10.3390/microorganisms11010175)
Supplement: Supplementary file 1 [file microorganisms-11-00175-s001.zip › 10_Abdulkadir_FC_MAG_Supplementary_file_8_Table_S6.pdf]

Combining Flow Cytometry and Metagenomics Improves Recovery of Metagenome-Assembled Genomes in a Cell Culture from Activated Sludge

Nafi'u Abdulkadir, Joao Pedro Saraiva, Florian Schattenberg, Rodolfo Brizola Toscan, Felipe Borim Correa, Hauke Harms, Susann Müller, Ulisses Nunes da Rocha

Supplementary file 8: Table S6. Comparison of relative abundances expressed in percentage of 16S rRNA genes operational taxonomic units (rOTUs) found in metagenome-assembled genome operational taxonomic units (gOTUs). The numbers indicate the relative abundances of gOTUs and rOTUs in the library. The dash (-) indicates rOTU was not found (below detection limit). (DG: Dominant sub-community; LA: Low abundant sub-community; OG: Outer gate sub-community; UC: Unsorted sub-community). The taxonomy of the gOTUs were defined using GTDB-tk v0.3.2 [21] and the rOTUs using EZbiocloud [34].

gOTU	rOTU	gOTUs sub-community				rOTUs sub-community				gOTU taxa	rOTU taxa
		DG	LA	OG	UC	DG	LA	OG	UC		
gOTU_01	rOTU_01	61.06	20.59	36.92	27.32	28.87	5.57	11.31	4.89	*Escherichia flexneri*	*Escherichia flexneri*
gOTU_02	rOTU_02	0.46	9.25	4.27	7.21	-	1.97	2.52	2.68	*Comamonas terrigena*	*Comamonas terrigena*
gOTU_03	rOTU_03	10.29	15.87	17.60	6.18	32.01	3.84	39.29	29.62	*Acinetobacter bouvetii*	*Acinetobacter bouvetii*
gOTU_04	rOTU_04	8.33	7.10	10.45	22.25	2.48	3.31	5.00	2.05	*Acinetobacter baumannii*	*Acinetobacter baumannii*
gOTU_05	-	2.29	1.65	1.40	2.21	-	-	-	-	*Sphingobacterium sp*	
gOTU_06	rOTU_05	0.59	1.24	3.21	9.43	0.34	0.78	1.11	7.84	*Empedobacter falsenii*	*Empedobacter falsenii*
gOTU_07	-	1.94	3.05	3.18	3.56	-	-	-	-	*Elizabethkingia ursingii*	

gOTU_08	rOTU_06	1.16	2.94	2.34	2.63	0.91	1.55	1.66	1.63	*Elizabethkingia miricola*	*Elizabethkingia miricola*
gOTU_09	rOTU_07	12.33	12.03	9.81	13.73	14.62	3.75	15.77	21.91	*Acinetobacter pittii*	*Acinetobacter pittii*
gOTU_10	rOTU_08	1.26	2.81	4.28	1.81	1.87	0.61	-	5.33	*Acinetobacter gerneri*	*Acinetobacter gerneri*
gOTU_11	-	0.30	23.47	6.53	3.67	-	-	-	-	*Variovorax sp.*	
-	rOTU_09						5.16	0.41	0.64		*Achromobacter anxifer*
-	rOTU_10							0.59			*Achromobacter insuavis*
-	rOTU_11						6.25	1.69	0.98		*Acidovorax antarcticus*
-	rOTU_12					0.14	0.07	4.89	0.45		*Acinetobacter bereziniae*
-	rOTU_13						12.09	1.57			*Acinetobacter gandensis*
-	rOTU_14							0.51			*Bacillus tropicus*
-	rOTU_15								1.66		*Brevundimonas olei*
-	rOTU_16						0.21	0.56	2.67		*Chryseobacterium artocarpi*
-	rOTU_17					0.04		0.07	0.96		*Chryseobacterium geocarposphaerae*
-	rOTU_18					5.60	31.55	4.07	7.94		*Citrobacter pasteurii*

-	rOTU_19						4.42	0.99		*Delftia acidovorans*
-	rOTU_20						1.05			*Diaphorobacter ruginosibacter*
-	rOTU_21						0.16	1.80		*Enterobacter hormaechei*
-	rOTU_22							0.35		*Klebsiella granulomatis*
-	rOTU_23					0.01	3.81	0.63		*Klebsiella huaxiensis*
-	rOTU_24					10.00	0.16			*Pseudomonas hunanensis*
-	rOTU_25						2.98	1.15	4.00	*Pseudomonas qingdaonensis*
-	rOTU_26					0.98	2.98	0.87	1.38	*Sphingobacterium multivorum*
-	rOTU_27					2.05	0.54	2.53	2.73	*Stenotrophomonas pavanii*
-	rOTU_28					0.08	2.03	0.25		*Stenotrophomonas terrae*
-	rOTU_29						5.16	0.41	0.64	*Moraxella osloensis*
*Detection limit		0.30	1.24	1.40	1.81	0.01	0.07	0.07	0.45	
*Average detection limit		**1.19**				**0.15**				
