# Supplementary material for: Combining Flow Cytometry and Metagenomics Improves Recovery of Metagenome-Assembled Genomes in a Cell Culture from Activated Sludge
Source: Microorganisms. 2023 Jan 10;11(1):175. doi: 10.3390/microorganisms11010175 (PMC9864227; doi:10.3390/microorganisms11010175)
Supplement: Supplementary file 1 [file microorganisms-11-00175-s001.zip › 11_Abdulkadir_FC_MAG_Supplementary_file_9_Table_S7.pdf]

Combining Flow Cytometry and Metagenomics Improves Recovery of Metagenome-Assembled Genomes in a Cell Culture from Activated Sludge

Nafi'u Abdulkadir, Joao Pedro Saraiva, Florian Schattenberg, Rodolfo Brizola Toscan, Felipe Borim Correa, Hauke Harms, Susann Müller, Ulisses Nunes da Rocha

Supplementary file 9: Table S7. Comparison of metagenome-assembled genome operational taxonomic units (gOTUs) and 16S rRNA genes operational taxonomic units (rOTUs) in the unsorted community (UC) and sorted sub-communities (dominant sub-community (DG), low abundant sub-community (LA), and outer sub-community (OG)). (✓ - recovered; × - not recovered). The taxonomy of gOTUs were assigned using GTDB-tk v0.3.2 [21] and rOTUs using EZbiocloud [34].

rOTUs ID	gOTUs ID	gOTUs				rOTUs				Taxonomy
		DG	LA	OG	UC	DG	LA	OG	UC	
rOTU_01	gOTU_01	✓	✓	✓	✓	✓	✓	✓	✓	*Escherichia flexneri*
rOTU_02	gOTU_02	×	✓	✓	✓	×	✓	✓	✓	*Comamonas terrigena*
rOTU_03	gOTU_03	✓	✓	✓	✓	✓	✓	✓	✓	*Acinetobacter bouvetii*
rOTU_04	gOTU_04	×	×	×	✓	✓	✓	✓	✓	*Acinetobacter baumannii*
-	gOTU_05	×	✓	✓	✓	×	×	×	×	*Sphingobacterium sp.*
rOTU_05	gOTU_06	×	✓	✓	✓	×	×	×	✓	*Empedobacter falsenii*
-	gOTU_07	✓	×	×	×	×	×	×	×	*Elizabethkingia ursingii*
rOTU_06	gOTU_08	×	✓	×	×	✓	✓	✓	✓	*Elizabethkingia miricola*
rOTU_07	gOTU_09	×	✓	×	×	✓	✓	✓	✓	*Acinetobacter pittii*

rOTU_08	gOTU_10	×	×	✓	×	✓	✓	×	✓	*Acinetobacter gerneri*
-	gOTU_11	×	✓	✓	✓	×	×	×	×	*Variovorax sp.*
rOTU_09	-	×	×	×	×	✓	✓	✓	×	*Achromobacter anxifer*
rOTU_10	-	×	×	×	×	×	×	✓	×	*Achromobacter insuavis*
rOTU_11	-	×	×	×	×	×	✓	×	×	*Acidovorax antarcticus*
rOTU_12	-	×	×	×	×	✓	✓	✓	✓	*Acinetobacter bereziniae*
rOTU_13	-	×	×	×	×	×	✓	✓	×	*Acinetobacter gandensis*
rOTU_14	-	×	×	×	×	×	×	✓	×	*Bacillus tropicus*
rOTU_15	-	×	×	×	×	×	×	×	✓	*Brevundimonas olei*
rOTU_16	-	×	×	×	×	×	✓	✓	✓	*Chryseobacterium artocarpi*
rOTU_17	-	×	×	×	×	✓	×	✓	✓	*Chryseobacterium geocarposphaerae*
rOTU_18	-	×	×	×	×	✓	✓	✓	✓	*Citrobacter pasteurii*
rOTU_19	-	×	×	×	×	×	✓	✓	×	*Delftia acidovorans*
rOTU_20	-	×	×	×	×	×	✓	×	×	*Diaphorobacter ruginosibacter*
rOTU_21	-	×	×	×	×	×	✓	✓	×	*Enterobacter hormaechei*
rOTU_22	-	×	×	×	×	×	×	✓	×	*Klebsiella granulomatis*
rOTU_23	-	×	×	×	×	✓	✓	✓	×	*Klebsiella huaxiensis*

rOTU_24	-	×	×	×	×	×	✓	×	×	*Pseudomonas hunanensis*
rOTU_25	-	×	×	×	×	×	✓	✓	✓	*Pseudomonas qingdaonensis*
rOTU_26	-	×	×	×	×	✓	✓	✓	✓	*Sphingobacterium multivorum*
rOTU_27	-	×	×	×	×	✓	✓	✓	✓	*Stenotrophomonas pavanii*
rOTU_28	-	×	×	×	×	✓	✓	✓	×	*Stenotrophomonas terrae*
rOTU_29	-	×	×	×	×	×	✓	✓	✓	*Moraxella osloensis*
